# Does an alternate-day modified fasting diet improve premenstrual syndrome symptoms and health-related quality of life in obese or overweight women with premenstrual syndrome? A randomized, controlled trial

**DOI:** 10.3389/fnut.2023.1298831

**Published:** 2024-01-10

**Authors:** Saeedeh Hosseini Hooshiar, Akram Yazdani, Sadegh Jafarnejad

**Affiliations:** ^1^Research Center for Biochemistry and Nutrition in Metabolic Diseases, Kashan University of Medical Sciences, Kashan, Iran; ^2^Department of Biostatistics and Epidemiology, School of Public Health, Kashan University of Medical Sciences, Kashan, Iran

**Keywords:** intermittent fasting, alternate-day modified fasting, calorie restriction, premenstrual syndrome, PMS, health-related quality of life, obesity, overweight

## Abstract

**Background:**

Premenstrual syndrome disorder (PMS) is a condition that affects health-related quality of life (HRQoL) and encompasses a variety of symptoms, including psychological, physical, and behavioral symptoms. Some evidence suggests that an increase in body mass index (BMI) can reduce both HRQoL and menstrual quality. This is because the body fat tissue can affect menstrual cycles by changing the estrogen/progesterone ratio. This study investigated the impact of two diets alternate-day modified fasting (ADMF) and daily calorie restriction (DCR) – on PMS syndrome and HRQoL.

**Methods:**

The study was a randomized controlled, open-label trial that lasted for 8 weeks and involved 60 obese/overweight women. Participants were recruited from the Health Service Centers of Kashan University of Medical Sciences using simple random sampling. The study compared the impact of the ADMF and DCR diets on HRQoL and PMS symptoms. Patients were classified based on their BMI and age and then allocated to either the intervention (ADMF) or control (DCR) group using a random numbers table. The study measured HRQoL, PMS severity, weight, BMI, body fat mass, waist circumference, fat-free mass, and skeletal muscle mass before and after the study. The study had an almost 18% dropout rate.

**Results:**

Significant improvements were observed in mood lability (*p* = 0.044) and expressed anger (*p* < 0.001) in relation to PMS symptoms. However, no significant differences were detected in the changes of other COPE subscales. The ADMF diet had a significant impact on the 12-item Short-Form Health Survey (SF-12) total score (*p* < 0.001) and physical function subscales (*p* = 0.006) as well as mental health (*p* < 0.001) when compared to the control diet. This implies that the ADMF diet increased both SF-12 total score and its subscales. The intervention led to improvements in HRQoL, physical function, and mental health. Additionally, significant improvements in BMI and weight were observed between the two groups pre- and post-study (*p* < 0.001). Anthropometric data, including body fat mass and waist circumference, showed a significant improvement (*p* < 0.001 and *p* = 0.029, respectively) before and after the study. However, there were no significant changes in fat-free mass (*p* = 0.936) and skeletal muscle mass (*p* = 0.841) between the two groups.

**Conclusion:**

The study suggested that ADMF can improve HRQoL, mood lability, and expressed anger. It also showed that ADMF can reduce waist circumference, weight, and body fat mass in obese/overweight women.

**Clinical trial registration:**

The Iranian Registry of Clinical Trials (IRCT20220522054958N1).

## Introduction

PMS is a common health issue among women of childbearing age, which greatly affects their quality (1). This disorder not only impacts women’s psychological health and social relationships but also reduces their work performance due to its physical and behavioral symptoms (2). PMS typically occurs during the luteal phase of the menstrual cycle (3). The prevalence of this disorder is 47.8% worldwide (4), while in Iran, it ranges from 33 to 48% (5). Women with PMS may experience various symptoms, including behavioral, emotional, and physical signs such as depression, isolation, irritability, and bloating (6). While the exact causes of PMS are still unclear, various factors are known to contribute to this condition (7). Age, family history, taking contraceptive pills, smoking, stress, BMI, exercise, and dietary habits are some of the factors that can lead to menstrual problems (8). Therefore, different strategies have been suggested to manage PMS, such as medications, specific supplements, dietary recommendations, psychological methods, and exercise. Research is ongoing to find more effective and safer treatments (9).

Dietary interventions can be safe and effective strategies for managing PMS (10). Research suggests that menstrual disorders are linked to obesity (11). In a study by Ju et al. (12), it was found that overweight/obese and underweight women suffer from menstrual disorders more than normal-weight women. High levels of prostaglandins have been associated with menstrual problems (13), and the levels of body fat can impact the menstrual cycle and normal ovulation, potentially leading to menstrual disorders (14). Obesity can also alter the function of neurotransmitters that affect progesterone and estrogen hormones (15). Studies have shown that every unit of increased BMI increases the risk of PMS by 3% (16). It is possible that abnormal menstruation and irregular ovulation can affect the estrogen/progesterone ratio, leading to an increase in prostaglandins and thus menstrual disorders (17).

Additionally, changes in the levels of progesterone and estrogen during the menstrual cycle may affect brain chemicals such as serotonin, which can influence mood (18). Research has also linked obesity and overweight to reduced quality of life in various populations (19). Quality of life refers to a subjective sense of overall wellbeing and satisfaction with life. HRQoL (health-related quality of life) is a tool that can help assess the functional impact of diseases and has high acceptability (20). Studies have found that the most common problems experienced by young women are related to menstruation (21).

Calorie restriction has been suggested as the primary treatment for overweight or obese individuals (22). However, patients have low adherence to common calorie restriction diets as they need to be followed daily (23). Intermittent fasting diets have been proposed as an alternative weight loss method and have been shown to improve metabolic health (24). Among various intermittent fasting methods, alternate-day modified fasting (ADMF) has been shown to reduce body weight by 3–7% in 2–3 months (25). However, it is unclear how the ADMF or calorie restriction diets affect body composition and anthropometric indices (22). Trepanowski et al. (22, 26) found that intermittent fasting can improve waist circumference more than usual calorie restriction diets but can decrease weight similarly to common diets. Some studies suggest that intermittent fasting diets decrease visceral fat and preserve muscle mass compared to daily calorie restriction diets (27). Other evidence shows that both intermittent fasting and daily calorie restriction diets increase muscle mass and do not change visceral fat tissue (22). Hutchison et al. (28) reported that intermittent fasting leads to higher decreases in body weight and fat tissue than common calorie restriction diets over 2 months. Some trials propose that attempting to lose weight increases the risk of menstrual disorders (29). Other evidence shows that menstrual disorders increase in women with a normal BMI who enter the BMI category of 25 or higher (12).

In a study by Anton et al. (30), it was found that a fasting diet not only led to significant improvements in HRQoL but also resulted in a small yet significant weight loss among participants. Another study by Etemadifar et al. (31) revealed that Ramadan fasting had a positive impact on the HRQoL of multiple sclerosis patients. Interestingly, the fasting diet was observed to enhance the quality of life independently of weight loss (32). Moreover, a few studies have emphasized that a fasting diet is safe and feasible and can boost the quality of life (33). However, Nugraha et al. (34) presented contradictory findings as their research showed that a Ramadan fasting diet did not affect HRQoL as compared to the control. Hence, due to the inconsistencies in the published results and the limitations of the studies conducted in this field, it is crucial to conduct further research to arrive at more conclusive findings. The primary objective of this trial was to evaluate the impact of the two diets, DCR and ADMF, on HRQoL and the severity of PMS. The findings of this trial can potentially be utilized to enhance dietary recommendations.

## Materials and methods

### Participants

This is a randomized, controlled, open-label trial. Participants were selected from the health centers of Kashan University of Medical Sciences considering the inclusion and exclusion criteria using a simple random sampling method. The inclusion criteria were women aged between 18 and 50 years, with a BMI equal to or greater than 25 and less than 40, menstrual bleeding lasting between 3 and 8 days, and normal menstrual cycles of 21–35 days. The participants also had PMS based on PSST (premenstrual symptoms screening tool) and agreed to comply with the study methods. The exclusion criteria included chronic disorders such as heart disease, diabetes, hypertension, digestive problems such as gastritis, peptic ulcer, and duodenal ulcer, breastfeeding, pregnancy, alcohol abuse, smoking habit, weight loss of more than 1 to 2 kg in the last month, nutritional supplements to lose weight, adherence to a special diet, and medication use in the last 2 months. Mental or psychiatric disorders such as depression, clinically diagnosed diseases such as infectious cancer, kidney, CVD, liver, neurological, endocrine, and gynecological diseases were also the exclusion criteria. In addition, having surgery in the last 6 months, enduring extreme stress during the trial, taking antidepressants and contraceptives, taking B6 supplements in the past 3 months, and not completing the questionnaire for 3 successive days and 5 non-successive days were other exclusion criteria.

### Study design

**Figure 1:**
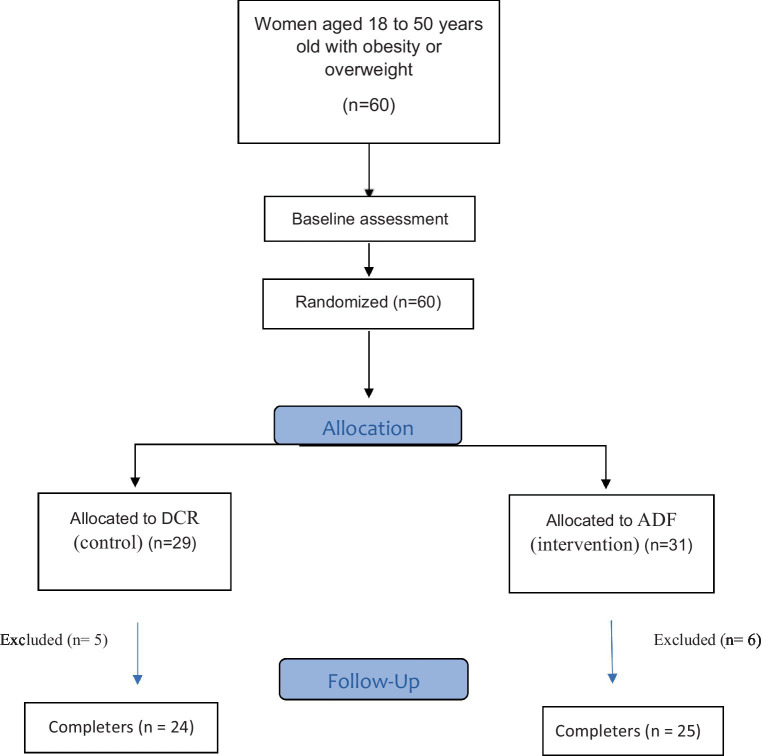
Flow chart of the intervention.

The flow diagram for the study is presented in Figure 1. A total of 60 eligible participants were sorted based on their BMI and age and then randomly assigned to either the “ADMF” (intervention) or “DCR” (control) groups after initial investigations at the start of the study. A statistician created the allocation sequences with a table of random numbers (35). Participants were assessed based on the eligibility criteria by a trained nutritionist and a medical doctor, who then included them in the trial. The trial protocol was explained by the nutritionist, who also obtained informed written consent and maintained contact with the patients via phone at their workplaces or homes throughout the trial.

All participants followed their prescribed diet for 8 weeks according to the group they were in and their energy requirements. The energy needs of patients were evaluated using the Mifflin equation (36). A professional dietician provided dietary counseling to patients. Individuals in the control and fasting groups were required to maintain their usual physical activity during the 8-week study period. Participants completed the food record questionnaire 3 days a week (2 days during the week and 1 day off) once every 2 weeks to ensure adherence to the prescribed diet (37). They were asked to complete food records during the week, one fast day, and one feast day. The food record was completed on a day off, whether it was a fast day or a feast day. All participants were taught how to fill in the questionnaires and select appropriate days to complete the questionnaires correctly. Using the home scale guide, the results of the forms were converted to grams and calculated by N4 software (The first Databank Inc.; Hearst Corporation) for Iranian foods. Then, the intake of macronutrients and calories was determined. Participants were considered adherent when the total macronutrients and calorie consumption were between 80 and 110% of the recommended amounts (38). The study researchers regularly made phone calls to participants to encourage them to adhere to their diets. The Ethics Committee of Kashan University of Medical Sciences approved this trial with the ethics code IR.KAUMS.MEDNT.REC.1401.003 and the Iranian Registry of Clinical Trials with the registration code IRCT20220522054958N1. No special side effects were observed during the study.

### ADMF diet

The ADMF diet consisted of alternating periods of feasting and fasting, starting at midnight every day. During fasting days, participants had a 75% calorie restriction, consuming only a quarter of their daily energy needs between 12:00 PM and 2:00 PM. They were allowed to drink water and other calorie-free drinks, with a daily limit of 400 mg of caffeine. On feasting days, participants consumed their entire daily calorie requirement. The diet was followed for 8 weeks, with a prescribed daily intake of 15% protein, 30% fat, and 55% carbohydrates. Participants were required to maintain their daily routine activities, and all had the same number of contacts with the dietician. The food was prepared at home by the individuals.

### DCR diet

During the study, a group of individuals (DCR) were allocated 63% of their daily calorie requirements as the control group. The participants were required to follow their prescribed diet for 8 weeks and cook all their meals at home. The macronutrient composition of the diet was 15% protein, 30% fat, and 55% carbohydrates. The participants were asked to maintain their regular physical activity routine, and all patients had the same number of contacts with the dietician.

### Assessment of variables

#### PSST questionnaire

To diagnose PMS, the PSST questionnaire was used (39). The questionnaire has 19 items and consists of two sections. The first section has 14 questions related to physical, behavioral, and psychological symptoms, while the second section evaluates the impact of these symptoms on patients’ lives. This section has five components. Patients were asked to report their symptoms during 5 days before their menstruation (40). Symptoms were rated on a scale of 1 to 4 (1: no symptoms and 4: severe symptoms).

To diagnose PMS, the following criteria were used: (I) a score of 3 or more in at least one of the four items related to feeling irritable, depressed, tearful, or tense; (II) a score of 3 or more in at least one of the five items related to interference with work performance, communication with friends and family, household tasks, or social life; (III) a score of 3 or more in at least four out of the first 14 items. Patients who did not meet these criteria were not included in the research (40).

#### Calendar of premenstrual experiences (COPE)

The study required women with PMS to record their symptoms daily for 3 months using the COPE assessment tool (41). The tool assesses 22 premenstrual symptoms, including 10 somatic and 12 behavioral symptoms, throughout the menstrual cycle (41, 42). Participants were required to rate the severity of their symptoms on a 0–3 point Likert scale (3: severe symptoms, 2: moderate symptoms, 1: mild symptoms, and 0: no symptoms) (43). At the end of each month, the scores for each symptom were totaled, and the PMS severity was determined. Scores less than 30% indicated mild PMS severity, scores between 30 and 50% indicated moderate severity, scores between 50 and 60% indicated severe severity, and scores over 60% indicated very severe PMS severity (44). Participants were required to complete this form for 3 months, i.e., 1 month before starting the study and 2 months during the study.

#### The 12-item short-form health survey (SF-12)

The Short Form Health Survey is a widely used public questionnaire that is applied globally (45). The SF-12 is a shorter version of the SF-36 questionnaire and consists of only 12 items (46, 47). Each item is scored on a 6-point Likert scale, with higher scores indicating better HRQoL (47, 48). To calculate the subscales of physical and mental health, the scores of certain items were added together. For the mental health subscale, scores of the social functioning, vitality, role restrictions due to emotional problems, and perceived mental health items were added together. For the physical health subscale, scores of physical functioning, bodily pain, role restrictions due to physical problems, and general health items were added together (49).

### Physical activity scale

The study measured the physical activity scale both before and after the experiment. To quantify the physical activity, the physical activity scale questionnaire based on MET (metabolic equivalents) was used which includes nine different levels (rest and sleep with a MET value of 0.9 to intense activity with a MET value greater than 6) (50). The physical activity scale quantifies the average daily amount of physical activity undertaken by individuals.

### Anthropometric data

Participants’ weight was evaluated by the Seca scale which had an accuracy of 0.1 kg, without shoes and wearing light clothes. Their height was measured with a stadiometer that had an accuracy of 0.5 cm, while standing barefoot. Body mass index (BMI) was measured accurately by dividing the participant’s weight (in kg) by the participant’s height squared (in meters). Waist circumference was measured, during exhaling, between the iliac crest and the lowest rib, during normal expiration, with an inelastic tape (51). Other anthropometric data, such as fat-free mass, skeletal muscle mass, waist-to-hip ratio, and body fat mass were calculated using InBody 770 (bioelectrical impedance analysis; InBody Co.). All anthropometric data were collected both before and after the trial.

#### Statistical assessment

To assess whether the data distribution is normal, we employed the Kolmogorov–Smirnov test. We used the chi-square test for qualitative data and the independent *t*-test for quantitative data to compare between groups (intervention and control). For comparing the within-group mean of quantitative variables, we used the paired *t*-test in normal conditions and the Wilcoxon test in non-normal conditions at the beginning and end of the trial. We used the Mann–Whitney test in non-normal conditions and the *t*-test in normal conditions to evaluate the mean changes between groups. We analyzed the data using the SPSS software (IBM, version 22) and expressed statistically significant results as a value of *p* of <0.05.

## Results

The flow diagram of this study can be seen in Figure 1. Initially, 60 women were randomly assigned to either the ADMF or calorie restriction groups. During the trial, six participants from the fasting group were excluded due to not adhering to the diet (*n* = 3), pregnancy (*n* = 1), and illness (*n* = 2). Similarly, five women from the control group were excluded due to not adhering to the diet (*n* = 4) and illness (*n* = 1). Eventually, 49 women finished the trial and were included in the final analysis. The demographic characteristics of the participants were displayed in Table 1, and no significant difference was observed between the two groups in terms of baseline characteristics, BMI (*p* = 0.535), age (*p* = 0.876), and physical activity (*p* = 0.106). Moreover, there were no significant changes in physical activity at the beginning and end of the study within the intervention (*p* = 0.353) and control (*p* = 0.601) groups (Table 1).

**Table 1:** General characteristics of study participants.

Characteristics	ADMF *n* = 25	DCR *n* = 24	Effect size	*p*-value
Age [Mean (SD)]	31.76 (8.10)	32.13 (8.15)	0.04^5^	0.876^1^
Marital [n (%)] Single Married	2 (8.0) 23 (92.0)	5 (20.8) 19 (79.2)	0.18^6^	0.192^2^
Child [n (%)] 0 1 2 3	8 (32.0) 6 (24.0) 9 (36.0) 2 (8.0)	9 (37.5) 4 (16.7) 8 (33.3) 3 (12.5)	0.11^6^	0.929^2^
Job [n (%)] Student Employee Housewife Unemployed	1 (4.0) 5 (20.0) 18 (72.0) 1 (4.0)	2 (8.3) 8 (33.3) 12 (50.0) 2 (8.3)	0.22^6^	0.541^2^
Economic [n (%)] Poor Average Good	0 (0.0) 18 (72.0) 7 (28.0)	2 (8.3) 20 (83.3) 2 (8.3)	0.31^6^	0.080^2^
Education [n (%)] Below diploma Diploma Bachelor and above	2 (8.0) 15 (60.0) 8 (32.0)	0 (0.0) 15 (62.5) 9 (37.5)	0.20^6^	0.606^2^
Physical activity [Mean (SD)] Baseline After 8 weeks *p*-value	26.57 (8.72) 26.35 (8.31) 0.353^4^	30.19 (6.46) 30.03 (6.67) 0.601^4^	0.04^5^ 0.04^5^	0.106^1^ 0.095^1^
BMI [Mean (SD)]	31.70 (3.11)	31.55 (3.72)	0.80^7^	0.535^3^

1 *p*-value: Independent samples *t*-test.

2 *p*-value: Fisher’s Exact test.

3 *p*-value: Mann- Whitney *U* test.

4 *p*-value: Paired samples test.

5 Effect size: Cohen’s d.

6 Effect size: Cramer’s V.

7 Effect size: Wendt formula, Effect sizes based on comparison of mean changes between two groups.

### COPE

In the ADMF group, a statistically significant decrease in mood lability was observed (*p* = 0.002) before and after the study. Additionally, there was a significant difference in the change in mood lability between the two groups (*p* = 0.044). The fasting group showed a significant change in expressed anger (*p* < 0.001) compared to the control group. However, there were no significant differences in the changes of other COPE subscales, such as oversensitivity, irritability, anxiety, crying easily, isolation, depression, dizziness, heart palpitation, nausea, poor concentration, forgetfulness, hot flash, headache, fatigue, increased appetite, food craving, acne, breast tenderness, swelling, and bloating, before and after the research between the control and intervention groups (Table 2).

**Table 2:** The COPE subscales, before and after the study.

Characteristics	Group	Baseline	After 8 weeks	*p*-value^1^	Effect size^2^	*p*-value^3^
Oversensitivity	ADMF *n* = 25	4.76 (2.74)	5.92 (3.30)	0.134	0.05	0.739
	DCR *n* = 24	4.70 (2.25)	5.87 (3.18)	0.196		
Mood lability	ADMF *n* = 25	10.08 (3.17)	8.36 (3.05)	0.002	0.33	0.044
	DCR *n* = 24	8.79 (2.91)	8.54 (3.92)	0.758		
Irritability	ADMF *n* = 25	10.80 (3.34)	10.64 (3.37)	0.892	0.03	0.840
	DCR *n* = 24	10.91 (3.93)	11.37 (4.29)	0.566		
Expressed anger	ADMF *n* = 25	12.12 (3.05)	10.56 (2.56)	0.001	0.61	<0.001
	DCR *n* = 24	10.62 (2.42)	11.50 (2.28)	0.054		
Anxiety	ADMF *n* = 25	5.52 (2.69)	6.44 (3.48)	0.211	0.06	0.694
	DCR *n* = 24	4.91 (2.14)	5.50 (3.07)	0.476		
Crying easily	ADMF *n* = 25	6.04 (2.63)	6.64 (3.40)	0.303	0.04	0.758
	DCR *n* = 24	4.50 (2.32)	5.58 (3.20)	0.130		
Isolation	ADMF *n* = 25	5.84 (2.65)	6.80 (3.40)	0.166	0.11	0.499
	DCR *n* = 24	4.95 (2.19)	5.33 (3.29)	0.700		
Depression	ADMF *n* = 25	5.44 (2.48)	6.80 (2.91)	0.046	0.03	0.832
	DCR *n* = 24	6.25 (2.26)	7.95 (3.27)	0.032		
Dizziness	ADMF *n* = 25	6.72 (3.16)	6.76 (2.93)	0.820	0.12	0.444
	DCR *n* = 24	9.08 (3.22)	10.16 (3.43)	0.247		
Heart palpitations	ADMF *n* = 25	9.00 (3.46)	9.04 (4.12)	0.976	0.03	0.823
	DCR *n* = 24	9.08 (3.02)	9.45 (3.82)	0.585		
Nausea	ADMF *n* = 25	1.92 (2.39)	0.56 (0.71)	0.006	0.01	0.927
	DCR *n* = 24	2.00 (2.41)	0.58 (0.71)	0.006		
Poor concentration	ADMF *n* = 25	1.68 (2.41)	0.44 (0.65)	0.013	0.01	0.910
	DCR *n* = 24	1.41 (1.58)	0.62 (0.76)	0.005		
Forgetfulness	ADMF *n* = 25	0.40 (0.64)	1.44 (2.10)	0.026	0.08	0.608
	DCR *n* = 24	0.54 (0.72)	158 (2.74)	0.158		
Hot flashes	ADMF *n* = 25	5.96 (2.55)	6.96 (3.23)	0.189	0.03	0.839
	DCR *n* = 24	5.33 (2.20)	6.75 (2.73)	0.066		
Headache	ADMF *n* = 25	0.24 (0.52)	0.84 (1.79)	0.110	0.07	0.641
	DCR *n* = 24	0.16 (0.38)	0.83 (1.23)	0.027		
Fatigue	ADMF *n* = 25	5.40 (2.23)	6.60 (3.75)	0.136	0.006	0.968
	DCR *n* = 24	5.45 (2.14)	6.70 (3.72)	0.109		
Increased appetite	ADMF *n* = 25	10.96 (3.58)	10.52 (3.45)	0.715	0.06	0.701
	DCR *n* = 24	10.41 (3.36)	10.83 (3.08)	0.817		
Food craving	ADMF *n* = 25	11.12 (3.85)	10.72 (3.38)	0.675	0.00	1.00
	DCR *n* = 24	11.08 (3.56)	10.29 (3.53)	0.666		
Acne	ADMF *n* = 25	10.56 (3.95)	9.84 (4.04)	0.585	0.06	0.716
	DCR *n* = 24	10.91 (3.30)	10.70 (3.82)	0.977		
Breast tenderness	ADMF *n* = 25	10.84 (3.67)	10.44 (3.39)	0.741	0.03	0.825
	DCR *n* = 24	10.08 (3.42)	10.12 (3.06)	0.919		
Swelling	ADMF *n* = 25	10.08 (3.98)	9.64 (3.93)	0.498	0.06	0.694
	DCR *n* = 24	10.41 (5.22)	9.62 (3.94)	0.354		
Bloating	ADMF *n* = 25	8.92 (3.80)	7.64 (3.70)	0.003	0.09	0.548
	DCR *n* = 24	8.79 (2.93)	7.87 (3.22)	0.022		

Values reported as Mean (SD).

1 *p*-value: Wilcoxon test.

2 Effect size: Wendt formula.

3 *p*-value: Mann–Whitney test.

Effect sizes based on comparison of mean changes between two groups.

### SF-12

Table 3 displays the SF-12 changes before and after the study. The ADMF resulted in significant improvements in the SF-12 total score (*p* < 0.001) as well as the physical function (*p* = 0.006) and mental health (*p* < 0.001) subscales compared to the control diet. This means that the intervention led to improved HRQoL, physical function, and mental health. Moreover, the percentage change in physical function, mental health, and SF-12 total score was higher in the intervention group than in the control group. The HRQoL questionnaire includes a specific subscale that focuses on participants’ physical health, specifically referring to illness and injury experienced in the past 30 days. Although Table 1 indicates similar levels of physical activity between the intervention and control groups, the divergent results observed in HRQoL and its subscales imply that the intervention group exhibited enhancements in both physical and mental wellbeing.

**Table 3:** The SF-12, physical function, and mental health before and after the study.

Characteristics	Group	Baseline	After 8 weeks	٪change	*p*-value	Effect size	*p*-value
Physical function	ADMF *n* = 25	9.36 (2.17)	10.72 (2.40)	14.53	0.009^1^	0.44^5^	0.006^2^
	DCR *n* = 24	9.75 (2.28)	9.91 (2.63)	1.64	0.296^1^		
Mental health	ADMF *n* = 25	12.44 (2.25)	14.20 (1.58)	14.15	<0.001^1^	0.58^5^	<0.001^2^
	DCR *n* = 24	12.75 (2.30)	11.66 (2.33)	−8.55	0.091^1^		
SF-12 total score	ADMF *n* = 25	21.80 (3.48)	24.92 (2.73)	14.31	<0.001^3^	1.41^6^	<0.001^4^
	DCR *n* = 24	22.50 (3.41)	21.58 (3.72)	−4.09	0.110^3^		

Values reported as Mean (SD).

1 *p*-value: Wilcoxon test.

2 *p*-value: Mann–Whitney test.

3 *p*-value: Paired *t*-test.

4 *p*-value: Independent *t*-test.

5 Effect size: Wendt formula.

6 Effect size: Cohen’s *d*, Effect sizes based on comparison of mean changes between two groups.

### Anthropometric indices

At the end of an 8-week intervention, the body weight and BMI of both control and intervention groups showed a significant decrease before and after the study (*p* < 0.001 and *p* < 0.001, respectively). Furthermore, the BMI and weight changes between the two groups were significant both before and after the study (*p* < 0.001). The intervention group had a higher percentage change in body weight compared to the control group (−6.68% vs. −3.72%). However, there were no significant changes in fat-free mass (*p* = 0.936) and skeletal muscle mass (*p* = 0.841) between the two groups. Other anthropometric data, including BFM (*p* < 0.001) and waist circumference (*p* = 0.029), showed significant differences at the beginning and end of the study between the two groups. Additionally, the percentage change of BFM and WC was higher in the fasting group than the control group (Table 4).

**Table 4:** The anthropometric indices, at baseline and after the 8-week.

Characteristics	Group	Baseline	After 8 weeks	٪change	*p*-value^1^	Effect size^2^	*p*-value^3^
Weight	ADMF *n* = 25	81.17 (12.73)	75.75 (12.12)	−6.68	<0.001	0.83	<0.001
	DCR *n* = 24	82.05 (13.35)	79.00 (12.82)	−3.72	<0.001		
BMI	ADMF *n* = 25	31.70 (3.11)	29.56 (3.08)	−6.75	<0.001	0.80	<0.001
	DCR *n* = 24	31.55 (3.72)	30.38 (3.68)	−3.71	<0.001		
FFM	ADMF *n* = 25	46.01 (6.21)	45.03 (6.38)	−2.13	<0.001	0.01	0.936
	DCR *n* = 24	46.75 (6.15)	45.70 (6.00)	−2.25	<0.001		
SMM	ADMF *n* = 25	25.24 (3.63)	24.56 (3.73)	−2.69	<0.001	0.03	0.841
	DCR *n* = 24	25.62 (3.72)	24.95 (3.49)	−2.62	<0.001		
BFM	ADMF *n* = 25	35.15 (8.44)	30.70 (8.08)	−12.66	<0.001	0.77	<0.001
	DCR *n* = 24	35.33 (9.01)	33.44 (8.86)	−5.35	<0.001		
WC	ADMF *n* = 25	102.98 (8.09)	98.62 (10.88)	−4.23	<0.001	0.35	0.029
	DCR *n* = 24	102.33 (12.87)	99.87 (12.72)	−2.40	<0.001		

Values reported as Mean (SD).

1 *p*-value: Wilcoxon test.

2 Effect size: Wendt formula.

3 *p*-value: Mann–Whitney test. Effect sizes based on comparison of mean changes between two groups. BMI, Body mass index; FFM, fat-free mass; SMM, skeletal muscle mass; BFM, body fat mass; WC, waist circumference.

## Discussion

Our trial has shown that following an ADMF diet for 8 weeks can lead to significant improvements in mood lability and reduced expression of anger. Mood swings can be caused by hormonal fluctuations of progesterone and estrogen, which in turn affect the levels of serotonin, dopamine, and γ-aminobutyric acid. These hormones can also impact the renin-angiotensin system. The periodic effect of progesterone and estrogen is also mentioned as the cause of some PMS symptoms such as bloating, weight gain, and swelling (52). However, studies have shown that levels of progesterone and estrogen do not have a significant change between women with PMS and healthy women (53). Therefore, it is unknown why some women suffer from PMS while others do not. Several studies have suggested that women with PMS have greater sensitivity to hormonal differences within the menstrual cycle (54). If post-menopausal patients who previously suffered from PMS are treated with progesterone, the PMS symptoms will recur in them (54).

Studies have shown that obesity affects the levels of progesterone and estrogen hormones by altering the function of neurotransmitters that regulate them (15). In an 8-week study, an ADMF diet was found to be effective in improving weight, BMI, BFM, and WC. These results were consistent with the findings of another study by Bhutani et al., which showed that ADMF reduced fat mass and weight more than DCR (25). Although both ADMF and DCR resulted in weight loss after 8 weeks, the fasting diet was more effective in reducing BMI and weight than the control. These results were consistent with the results of previous studies, such as Razavi et al. (55) and Johnson et al. (56). Participants in ADMF groups have been found to be more compliant with their prescribed diet compared to those on DCR (57). In traditional diets, food consumption is restricted every day, (58) whereas in ADMF diets, calorie intake is restricted every other day, which leads to greater adherence to the diet (57). This adherence to fasting diets leads to more weight loss compared to traditional diets. In the fasting diet, a significant part of decreased weight is associated with the reduction of fat tissue, while fat-free mass is commonly retained (59). Since participants in ADMF diets have a fast for 3–4 days a week, greater weight loss is often seen compared to the control (60). During fasting hours, the body uses ketones and fat as the primary sources of energy, leading to a reduction in fat tissue and body weight (61, 62). People on fasting diets have reported a decrease in appetite during fasting. The change in appetite may be due to the change in appetite-regulating hormones, such as an increase in adiponectin levels and a decrease in leptin and resistin levels, as seen in animal experiments (63).

Our study found that following the ADMF diet resulted in a significant improvement in the SF-12 total score, as well as the physical function and mental health subscales, when compared to the control diet. Previous studies assessing HRQoL using the SF-12 in various populations have suggested that PMS symptoms can have an impact on HRQoL, including mental and physical subscales (64). In our study, the HRQoL and subscale scores of the ADMF group were better than those of the CRD group, which was likely due to the fasting group’s improved physical and mental health. This result was consistent with previous findings that have reported an increase in quality of life and a decrease in fatigue in gynecological cancer patients (65) as well as other studies indicating that intermittent fasting resulted in an improvement in quality of life and physical and mental fatigue in healthy individuals (66).

One of the limitations of our trial was that the assessment of premenstrual syndrome and quality of life was based on self-reported forms, which could cause inaccuracies. Additionally, this study did not investigate the long-term outcomes of the ADMF diet. Our study had a notable limitation; in that, we performed a per-protocol analysis. As a result, we recommend that future studies also consider alternative analyses, such as intention-to-treat analysis, to enhance the robustness of the findings. By doing so, a more comprehensive understanding of the research outcomes can be achieved. To assess adherence to the recommended diet, we utilized food record forms. It is important to note that collecting food records for only 3 days a week may not provide a comprehensive representation of participants’ dietary intake for the entire week. However, this approach was deliberately chosen to minimize the burden on participants and ensure compliance with the study protocol. Additionally, we conducted regular phone interviews with patients throughout the trial period as an additional measure of control and to enhance data accuracy. Furthermore, this was the first research to assess the effect of an ADMF diet on PMS symptoms. This trial assessed an extensive range of health outcomes, including PMS severity, multiple anthropometric indices, and health-related quality of life measures.

## Conclusion

Weight loss is a promising method for controlling PMS, and ADMF has been proposed as an effective diet for weight loss and improving metabolic status. Our study found that ADMF is a safe diet for weight loss and BMI management in overweight and obese women. It could also improve HRQoL, mood lability, and expressed anger. However, further studies are needed to establish causality and generalize the findings to other populations.

## Data availability statement

The raw data supporting the conclusions of this article will be made available by the authors, without undue reservation.

## Ethics statement

The studies involving humans were approved by Ethics Committee of Kashan University of Medical Sciences. The studies were conducted in accordance with the local legislation and institutional requirements. The participants provided their written informed consent to participate in this study.

## Author contributions

SH: Data curation, Methodology, Writing – original draft. AY: Data curation, Formal analysis, Methodology, Software, Validation, Writing – review & editing. SJ: Conceptualization, Investigation, Methodology, Project administration, Supervision, Validation, Writing – review & editing.

## References

[1] Steiner M Born L. Diagnosis and treatment of premenstrual dysphoric disorder: an update. Int Clin Psychopharmacol. (2000) 15 Suppl 3:S5–S17. doi: 10.1016/S0140-6736(00)02749-5, PMID: 11195269

[2] Maharaj S Trevino K. A comprehensive review of treatment options for premenstrual syndrome and premenstrual dysphoric disorder. J Psychiatr Pract. (2015) 21:334–50. doi: 10.1097/PRA.0000000000000099 26352222

[3] Singh B Berman B Simpson R Annechild A. Incidence of premenstrual syndrome and remedy usage: a national probability sample study. Altern Ther Health Med. (1998) 4:75–9. PMID: 9581324

[4] Direkvand-Moghadam A Sayehmiri K Delpisheh A Kaikhavandi S. Epidemiology of premenstrual syndrome (PMS)-a systematic review and meta-analysis study. J Clin Diagn Res. (2014) 8:106. doi: 10.7860/JCDR/2014/8024.4021 24701496 PMC3972521

[5] Maleki F Pourshahbaz A Asadi A Yoosefi A. The impact of premenstrual disorders on health-related quality of life (HRQOL). practice in clinical psychology. (2014) 2:77–84.

[6] Halbreich U O’Brien P Eriksson E Bäckström T Yonkers KA Freeman EW. Are there differential symptom profiles that improve in response to different pharmacological treatments of premenstrual syndrome/premenstrual dysphoric disorder? CNS Drugs. (2006) 20:523–47. doi: 10.2165/00023210-200620070-00001, PMID: 16800714

[7] Nworie KM Aluh DO Onyekwum CA. Assessment of premenstrual syndrome among female students in Southeast Nigeria. J Obstetr Gynecol Investig. (2018) 1:55–61. doi: 10.5114/jogi.2018.79426

[8] Rafique N Al-Sheikh MH. Prevalence of menstrual problems and their association with psychological stress in young female students studying health sciences. Saudi Med J. (2018) 39:67–73. doi: 10.15537/smj.2018.1.21438, PMID: 29332111 PMC5885123

[9] Freeman EW. Therapeutic management of premenstrual syndrome. Expert Opin Pharmacother. (2010) 11:2879–89. doi: 10.1517/14656566.2010.509344 20687778

[10] Kia AS Amani R Cheraghian B. The association between the risk of premenstrual syndrome and vitamin D, calcium, and magnesium status among university students: a case control study. Health Promot Perspect. (2015) 5:225–30. doi: 10.15171/hpp.2015.027, PMID: 26634201 PMC4667262

[11] Snehalata T Mahesh M. Relationship between body mass composition and primary dysmenorrhea. Indian J Physiother Occup Ther. (2016) 10:76–81. doi: 10.5958/0973-5674.2016.00017.4

[12] Ju H Jones M Mishra GD. A U-shaped relationship between body mass index and dysmenorrhea: a longitudinal study. PLoS One. (2015) 10:e0134187. doi: 10.1371/journal.pone.0134187, PMID: 26218569 PMC4517870

[13] Chan W Dawood MY Fuchs F. Prostaglandins in primary dysmenorrhea: comparison of prophylactic and nonprophylactic treatment with ibuprofen and use of oral contraceptives. Am J Med. (1981) 70:535–41. doi: 10.1016/0002-9343(81)90576-3, PMID: 7011011

[14] Frisch RE. 1 the right weight: body fat, menarche and ovulation. Baillieres Clin Obstet Gynaecol. (1990) 4:419–39. doi: 10.1016/S0950-3552(05)80302-5, PMID: 2282736

[15] Sutariya S Talsania N Shah C Patel M. An interventional study (calcium supplementation & health education) on premenstrual syndrome-effect on premenstrual and menstrual symptoms. Nat J Commun Med. (2011) 2:100–4.

[16] Masho SW Adera T South-Paul J. Obesity as a risk factor for premenstrual syndrome. J Psychosom Obstet Gynecol. (2005) 26:33–9. doi: 10.1080/01443610400023049, PMID: 15962720

[17] Zahradnik H Breckwoldt M. Contribution to the pathogenesis of dysmenorrhea. Arch Gynecol. (1984) 236:99–108. doi: 10.1007/BF02134006, PMID: 6596911

[18] Jahanfar S Lye M-S Krishnarajah IS. The heritability of premenstrual syndrome. Twin Res Hum Genet. (2011) 14:433–6. doi: 10.1375/twin.14.5.433, PMID: 21962135

[19] Kolotkin RL Andersen JR. A systematic review of reviews: exploring the relationship between obesity, weight loss and health-related quality of life. Clin Obes. (2017) 7:273–89. doi: 10.1111/cob.12203, PMID: 28695722 PMC5600094

[20] Sahin S Ozdemir K Unsal A. Evaluation of premenstrual syndrome and quality of life in university students. J Pak Med Assoc. (2014) 64:915–22. PMID: 25252518

[21] Taşçı K. Evaluation of nursing students’ premenstrual symptoms. TAF Preven Med Bull. (2006) 5:434–43.

[22] Trepanowski JF Kroeger CM Barnosky A Klempel M Bhutani S Hoddy KK. Effects of alternate-day fasting or daily calorie restriction on body composition, fat distribution, and circulating adipokines: secondary analysis of a randomized controlled trial. Clin Nutr. (2018) 37:1871–8. doi: 10.1016/j.clnu.2017.11.018, PMID: 29258678 PMC5988907

[23] Moreira EAM Most M Howard J Ravussin E. Dietary adherence to long-term controlled feeding in a calorie-restriction study in overweight men and women. Nutr Clin Pract. (2011) 26:309–15. doi: 10.1177/0884533611405992, PMID: 21586416 PMC4830337

[24] Hoddy KK Marlatt KL Çetinkaya H Ravussin E. Intermittent fasting and metabolic health: from religious fast to time-restricted feeding. Obesity. (2020) 28:S29–37. doi: 10.1002/oby.22829 32700827 PMC7419159

[25] Bhutani S Klempel MC Kroeger CM Trepanowski JF Varady KA. Alternate day fasting and endurance exercise combine to reduce body weight and favorably alter plasma lipids in obese humans. Obesity. (2013) 21:1370–9. doi: 10.1002/oby.20353, PMID: 23408502

[26] Trepanowski J Kroeger C Klempel M Calvo Y Varady K. Alternateday fasting versus daily calorie restriction for weight loss and cardio-protection (120.6). FASEB J. (2014) 28:120.6. doi: 10.1096/fasebj.28.1_supplement.120.6

[27] Gotthardt JD Verpeut JL Yeomans BL Yang JA Yasrebi A Roepke TA. Intermittent fasting promotes fat loss with lean mass retention, increased hypothalamic norepinephrine content, and increased neuropeptide Y gene expression in diet-induced obese male mice. Endocrinology. (2016) 157:679–91. doi: 10.1210/en.2015-1622, PMID: 26653760 PMC4733124

[28] Hutchison AT Liu B Wood RE Vincent AD Thompson CH O’Callaghan NJ. Effects of intermittent versus continuous energy intakes on insulin sensitivity and metabolic risk in women with overweight. Obesity. (2019) 27:50–8. doi: 10.1002/oby.22345, PMID: 30569640

[29] Hailemeskel S Demissie A Assefa N. Primary dysmenorrhea magnitude, associated risk factors, and its effect on academic performance: evidence from female university students in Ethiopia. Int J Women's Health. (2016) 8:489–96. doi: 10.2147/IJWH.S112768 27695366 PMC5034908

[30] Anton SD Lee SA Donahoo WT McLaren C Manini T Leeuwenburgh C. The effects of time restricted feeding on overweight, older adults: a pilot study. Nutrients. (2019) 11:1500. doi: 10.3390/nu11071500, PMID: 31262054 PMC6682944

[31] Etemadifar M Sayahi F Alroughani R Toghianifar N Akbari M Nasr Z. Effects of prolonged fasting on fatigue and quality of life in patients with multiple sclerosis. Neurol Sci. (2016) 37:929–33. doi: 10.1007/s10072-016-2518-9 26994616

[32] Kesztyüs D Fuchs M Cermak P Kesztyüs T. Associations of time-restricted eating with health-related quality of life and sleep in adults: a secondary analysis of two pre-post pilot studies. BMC Nutr. (2020) 6:76–8. doi: 10.1186/s40795-020-00402-2, PMID: 33327959 PMC7745395

[33] Choi IY Piccio L Childress P Bollman B Ghosh A Brandhorst S. A diet mimicking fasting promotes regeneration and reduces autoimmunity and multiple sclerosis symptoms. Cell Rep. (2016) 15:2136–46. doi: 10.1016/j.celrep.2016.05.009, PMID: 27239035 PMC4899145

[34] Nugraha B Ghashang SK Hamdan I Gutenbrunner C. Effect of Ramadan fasting on fatigue, mood, sleepiness, and health-related quality of life of healthy young men in summer time in Germany: a prospective controlled study. Appetite. (2017) 111:38–45. doi: 10.1016/j.appet.2016.12.030, PMID: 28027907

[35] Wilcox S Sharpe PA Parra-Medina D Granner M Hutto B. A randomized trial of a diet and exercise intervention for overweight and obese women from economically disadvantaged neighborhoods: sisters taking action for real success (STARS). Contemp Clin Trials. (2011) 32:931–45. doi: 10.1016/j.cct.2011.08.003, PMID: 21864718 PMC3204793

[36] Mifflin MD St Jeor ST Hill LA Scott BJ Daugherty SA Koh YO. A new predictive equation for resting energy expenditure in healthy individuals. Am J Clin Nutr. (1990) 51:241–7. doi: 10.1093/ajcn/51.2.241 2305711

[37] Parvaresh A Razavi R Abbasi B Yaghoobloo K Hassanzadeh A Mohammadifard N. Modified alternate-day fasting vs. calorie restriction in the treatment of patients with metabolic syndrome: a randomized clinical trial. Complement Ther Med. (2019) 47:102187. doi: 10.1016/j.ctim.2019.08.021, PMID: 31779987

[38] Razo-Olvera D Mabel F Martin-Vences AJ Brito-Córdova GX Elías-López D Landa-Anell MV. Primary barriers of adherence to a structured nutritional intervention in patients with dyslipidemia. Nutrients. (2021) 13:1744. doi: 10.3390/nu13061744, PMID: 34063795 PMC8223790

[39] Steiner M Macdougall M Brown E. The premenstrual symptoms screening tool (PSST) for clinicians. Arch Womens Ment Health. (2003) 6:203–9. doi: 10.1007/s00737-003-0018-4 12920618

[40] del Mar Fernández M Regueira-Méndez C Takkouche B. Psychological factors and premenstrual syndrome: a Spanish case-control study. PLoS One. (2019) 14:e0212557. doi: 10.1371/journal.pone.0212557 30840651 PMC6402625

[41] Mortola J Girton L Beck L Yen S. Diagnosis of premenstrual syndrome by a simple, prospective, and reliable instrument: the calendar of premenstrual experiences. Obstet Gynecol. (1990) 76:302–7. PMID: 2371035

[42] Mortola J Girton L Fischer U. Successful treatment of severe premenstrual syndrome by combined use of gonadotropin-releasing hormone agonist and estrogen/progestin. J Clin Endocrinol Metabol. (1991) 72:252–252F. doi: 10.1210/jcem-72-2-252, PMID: 1846868

[43] Feuerstein M Shaw WS. Measurement properties of the calendar of premenstrual experience in patients with premenstrual syndrome. J Reprod Med. (2002) 47:279–89. PMID: 12012879

[44] Khajehei M Abdali K Parsanezhad ME Tabatabaee HR. Effect of treatment with dydrogesterone or calcium plus vitamin D on the severity of premenstrual syndrome. Int J Gynecol Obstet. (2009) 105:158–61. doi: 10.1016/j.ijgo.2009.01.016 19232611

[45] Bullinger M. German translation and psychometric testing of the SF-36 health survey: preliminary results from the IQOLA project. Soc Sci Med. (1995) 41:1359–66. doi: 10.1016/0277-9536(95)00115-N, PMID: 8560303

[46] Ware JE Jr Kosinski M Keller SD. A 12-item short-form health survey: construction of scales and preliminary tests of reliability and validity. Med Care. (1996) 34:220–33. doi: 10.1097/00005650-199603000-00003 8628042

[47] Ware J Kosinski M Keller S. SF-36 physical and mental health summary scales: a user’s manual. Boston, MA: Health Assessment Lab (1994).

[48] Lim LL Seubsman S-a Sleigh A. Thai SF-36 health survey: tests of data quality, scaling assumptions, reliability and validity in healthy men and women. Health Qual Life Outcomes. (2008) 6:52–9. doi: 10.1186/1477-7525-6-52, PMID: 18634552 PMC2515296

[49] Montazeri A Vahdaninia M Mousavi SJ Omidvari S. The Iranian version of 12-item short form health survey (SF-12): factor structure, internal consistency and construct validity. BMC Public Health. (2009) 9:1–10. doi: 10.1186/1471-2458-9-341, PMID: 19758427 PMC2749829

[50] Aadahl M Jørgensen T. Validation of a new self-report instrument for measuring physical activity. Med Sci Sports Exerc. (2003) 35:1196–202. doi: 10.1249/01.MSS.0000074446.02192.14 12840642

[51] World Health Organization. Waist circumference and waist-hip ratio: report of a WHO expert consultation. Geneva: World Health Organization (2011).

[52] Davis C Sloan M Tang C. Premenstrual distress among Caucasian, African-American and Chinese women. J Women’s Health Care. (2014) 3:181. doi: 10.4172/2167-0420.1000181

[53] Potter J Bouyer J Trussell J Moreau C. Premenstrual syndrome prevalence and fluctuation over time: results from a French population-based survey. J Women's Health. (2009) 18:31–9. doi: 10.1089/jwh.2008.0932, PMID: 19105683 PMC3196060

[54] Dennerstein L Lehert P Heinemann K. Global study of women's experiences of premenstrual symptoms and their effects on daily life. Menopause Int. (2011) 17:88–95. doi: 10.1258/mi.2011.011027, PMID: 21903712

[55] Razavi R Parvaresh A Abbasi B Yaghoobloo K Hassanzadeh A Mohammadifard N. The alternate-day fasting diet is a more effective approach than a calorie restriction diet on weight loss and HS-CRP levels. Int J Vitam Nutr Res. (2021) 91:242–50. doi: 10.1024/0300-9831/a000623, PMID: 32003649

[56] Johnson JB Summer W Cutler RG Martin B Hyun D-H Dixit VD. Alternate day calorie restriction improves clinical findings and reduces markers of oxidative stress and inflammation in overweight adults with moderate asthma. Free Radic Biol Med. (2007) 42:665–74. doi: 10.1016/j.freeradbiomed.2006.12.005, PMID: 17291990 PMC1859864

[57] Varady KA Hellerstein MK. Alternate-day fasting and chronic disease prevention: a review of human and animal trials. Am J Clin Nutr. (2007) 86:7–13. doi: 10.1093/ajcn/86.1.7, PMID: 17616757

[58] Malik VS Hu FB. Popular weight-loss diets: from evidence to practice. Nat Clin Pract Cardiovasc Med. (2007) 4:34–41. doi: 10.1038/ncpcardio0726 17180148

[59] Alhamdan B Garcia-Alvarez A Alzahrnai A Karanxha J Stretchberry D Contrera K. Alternate-day versus daily energy restriction diets: which is more effective for weight loss? A systematic review and meta-analysis. Obes Sci Pract. (2016) 2:293–302. doi: 10.1002/osp4.52, PMID: 27708846 PMC5043510

[60] Barnosky AR Hoddy KK Unterman TG Varady KA. Intermittent fasting vs daily calorie restriction for type 2 diabetes prevention: a review of human findings. Transl Res. (2014) 164:302–11. doi: 10.1016/j.trsl.2014.05.013 24993615

[61] El Ati J Beji C Danguir J. Increased fat oxidation during Ramadan fasting in healthy women: an adaptative mechanism for body-weight maintenance. Am J Clin Nutr. (1995) 62:302–7. doi: 10.1093/ajcn/62.2.302, PMID: 7625336

[62] De Cabo R Mattson MP. Effects of intermittent fasting on health, aging, and disease. N Engl J Med. (2019) 381:2541–51. doi: 10.1056/NEJMra1905136, PMID: 31881139

[63] Varady KA Allister CA Roohk DJ Hellerstein MK. Improvements in body fat distribution and circulating adiponectin by alternate-day fasting versus calorie restriction. J Nutr Biochem. (2010) 21:188–95. doi: 10.1016/j.jnutbio.2008.11.001 19195863

[64] Yang M Wallenstein G Hagan M Guo A Chang J Kornstein S. Burden of premenstrual dysphoric disorder on health-related quality of life. J Women’s Health. (2008) 17:113–21. doi: 10.1089/jwh.2007.0417, PMID: 18240988

[65] Bauersfeld SP Kessler CS Wischnewsky M Jaensch A Steckhan N Stange R. The effects of short-term fasting on quality of life and tolerance to chemotherapy in patients with breast and ovarian cancer: a randomized cross-over pilot study. BMC Cancer. (2018) 18:1–10. doi: 10.1186/s12885-018-4353-2 29699509 PMC5921787

[66] Anic K Schmidt MW Furtado L Weidenbach L Battista MJ Schmidt M. Intermittent fasting—short-and long-term quality of life, fatigue, and safety in healthy volunteers: a prospective, clinical trial. Nutrients. (2022) 14:4216. doi: 10.3390/nu14194216, PMID: 36235868 PMC9571750

